# Evaluation of olive processing wastes in terms of zero discharge and green energy production

**DOI:** 10.1038/s41598-025-27427-6

**Published:** 2025-11-12

**Authors:** Arzu Teksoy, Mehmet Şen

**Affiliations:** 1 Faculty of Engineering, Department of Environmental Engineering, Bursa Uludağ University, Nilüfer, Bursa, 16059 Turkey; 2 Marmarabirlik (Head Office & Integrated Plant), No. 682, İzmir Road, Başköy Neighborhood, Nilüfer, Bursa, Turkey

**Keywords:** Olive processing waste, Zero discharge, Biomass gasification, Organic rankine cycle, Biochar, Sustainable energy, Energy science and technology, Engineering, Environmental sciences

## Abstract

Olive oil production generates large volumes of solid and liquid waste with high organic load, acidity, and toxic phenolic compounds. Conventional disposal methods cause serious environmental problems such as groundwater contamination, greenhouse gas emissions, and inefficient use of resources. This study investigates a sustainable management approach for olive processing waste based on the zero discharge principle, integrating gasification and organic rankine cycle (ORC) systems for green energy production. The system operates with a biomass feed rate of 440 kg/h, produces 60 kg/h of biochar, and is powered by a thermal input of 2000 kWh_th_. The ORC unit operates at 15% electrical efficiency, generating 240 kW of electricity and 1360 kW of thermal energy. Mass and energy balance analyses show that nearly 100% of the waste can be converted into valuable outputs. Water recovery from olive mill wastewater was achieved through thermal evaporation. The biochar by-product can be utilized as a soil amendment or fuel additive. Economic feasibility analyses based on cost data demonstrate that profitability depends on electricity sale prices and the conversion of biochar into value-added products. A comparison of different biomass feedstocks reveals variability in cost but no significant difference in energy conversion efficiency. This research presents a viable, circular economy-oriented model for the olive oil industry in terms of sustainable waste valorization, water recovery, and renewable energy production.

## Introduction

Olive oil production in Mediterranean countries generates a significant amount of waste. In regions where olive oil is extensively produced, large quantities of olive pomace (solid waste) are formed. On average, the three-phase extraction system yields approximately 0.6 tons of solid waste (pomace) and 1.5 m³ of olive mill wastewater (OMW) per ton of olive oil produced. Globally, such wastes amount to nearly 40 million tons annually, with 10–30 million m³ being wastewater^1^.

The treatment of OMW is a common and pressing issue in many European and Mediterranean countries. OMW includes plant water from the olive fruit, washing water, process water, dissolved compounds from olive pulp, and residual oil. This dark-colored effluent contains organic substances such as sugars, organic acids, polyalcohols, pectins, colloids, tannins, and lipids. Its high content of BOD, COD, and phenolic compounds makes its treatment complex and costly. Moreover, OMW is phytotoxic and exhibits selective antimicrobial effects due to its polyphenol content^2^.

The uncontrolled discharge of OMW leads to serious environmental problems such as discoloration of natural waters, threats to aquatic ecosystems, contamination of surface and groundwater resources, soil degradation, phytotoxicity, and foul odor generation^1^. Due to high treatment costs and technical difficulties, olive mills often resort to low-cost but environmentally unsound disposal methods. For instance, OMW is commonly stored in open lagoons or directly discharged onto land or into streams^3^. Olive pomace is typically burned as fuel. While these methods are economical, they create additional environmental issues: dried crusts on lagoon surfaces hinder evaporation, leading to groundwater pollution and pest outbreaks, while methane and other greenhouse gases are emitted during anaerobic decomposition. Uncontrolled burning of solid waste contributes to air pollution and inefficient use of energy potential^4^.

Numerous OMW treatment technologies have been proposed, including lagooning or land irrigation^5^, co-composting^6^, physicochemical processes (flocculation, coagulation^7,8^, filtration^9,10^, open evaporation ponds^11,12^, electrocoagulation^13^, and membrane techniques such as ultrafiltration/reverse osmosis^14,15^. However, limitations exist for each method, indicating that no single universal solution currently exists for OMW treatment^16^. As a result, current waste management practices are neither environmentally nor economically sustainable. Many small-scale olive oil producers struggle to afford the high cost of waste disposal. Increasing environmental awareness and regulatory pressure make it imperative to develop alternative sustainable solutions. Within the framework of the circular economy, it is possible to transform waste into valuable resources.

The growing global energy demand and environmental impacts of fossil fuels have made the shift toward renewable and sustainable energy sources inevitable. Utilizing agricultural and industrial waste for energy production not only facilitates waste management but also supports the development of alternative energy sources. In this context, olive pomace, which is rich in organic matter, cellulose, hemicellulose, and lignin, emerges as a valuable biomass source for energy production^1^. The traditional disposal methods used by small and medium-sized producers—such as use as animal feed, composting, land spreading, or open burning—may appear inexpensive in the short term, but they result in long-term environmental and economic drawbacks. Moreover, many of these methods are now restricted or prohibited under environmental regulations in several countries, further increasing disposal costs and creating a need for alternatives.

The valorization of olive oil processing wastes as energy sources has gained increasing attention. Thermochemical conversion technologies, particularly gasification and pyrolysis, reduce environmental burdens while creating economic value. These methods convert organic wastes into valuable products such as syngas, bio-oil, and carbon-rich biochar. During slow pyrolysis, a significant portion of the carbon content in the feedstock is retained in the biochar, while energy-rich syngas and bio-oil are produced^17^. Traditional disposal methods fail to recover this energy potential. Gasification and ORC systems represent innovative technologies that can be integrated to convert waste into energy and useful products. These integrated systems are particularly relevant in the context of sustainable waste management^4,18^.

Gasification is especially effective for biomass with low moisture content; therefore, the homogenization (e.g., grinding or pelletizing) of olive pomace with typically 40–65% moisture improves gasification efficiency. Its Lower Heating Value (LHV) of 16–20 MJ/kg and high volatile matter content (70–80%) make it suitable for gasification^19,20^. In a study by Topal et al.^21^, the combustion performance of olive pomace from different regions in a fluidized bed furnace yielded efficiencies between 82.24% and 98.65%. Compared to coal, olive pomace exhibited lower residual carbon content. Flue gas analyses showed almost zero SO₂ and decreasing levels of CO and C_m_H_n_ with increased air supply^22,23^. Combustion experiments using olive pomace particles smaller than 0.2 mm in a vertical tube furnace achieved 82% combustion efficiency, 69% thermal efficiency, and a maximum flame temperature of 975 °C. Aguado et al.^24^ proposed an on-site compact gasification system for converting wet pomace into energy, highlighting its effectiveness and sustainability.

Similarly, Lajili et al.^25^ explored the energy conversion of agro-pellets derived from olive oil industry waste through fast pyrolysis and steam gasification. Fast pyrolysis yielded high amounts of bio-oil and biochar, while steam gasification produced hydrogen- and CO-rich syngas. Ducom et al.^26^ evaluated the physicochemical and thermal properties of pomace samples from various countries and concluded that properly dried and pelletized pomace is suitable for gasification, and the syngas produced can be used for both electricity and biofuel generation.

Most previous studies have focused on combustion characteristics, gas composition, and energy yield of single thermochemical processes. However, a comprehensive system based on the zero discharge principle for complete valorization of olive oil processing waste has not been addressed. Moreover, economic feasibility has rarely been explored. Therefore, this study evaluates an integrated system based on the zero discharge principle that transforms olive processing waste into useful outputs, and examines its technical performance, environmental benefits, and economic viability.

## Methodology

### Wastewater treatment

**Fig. 1:**
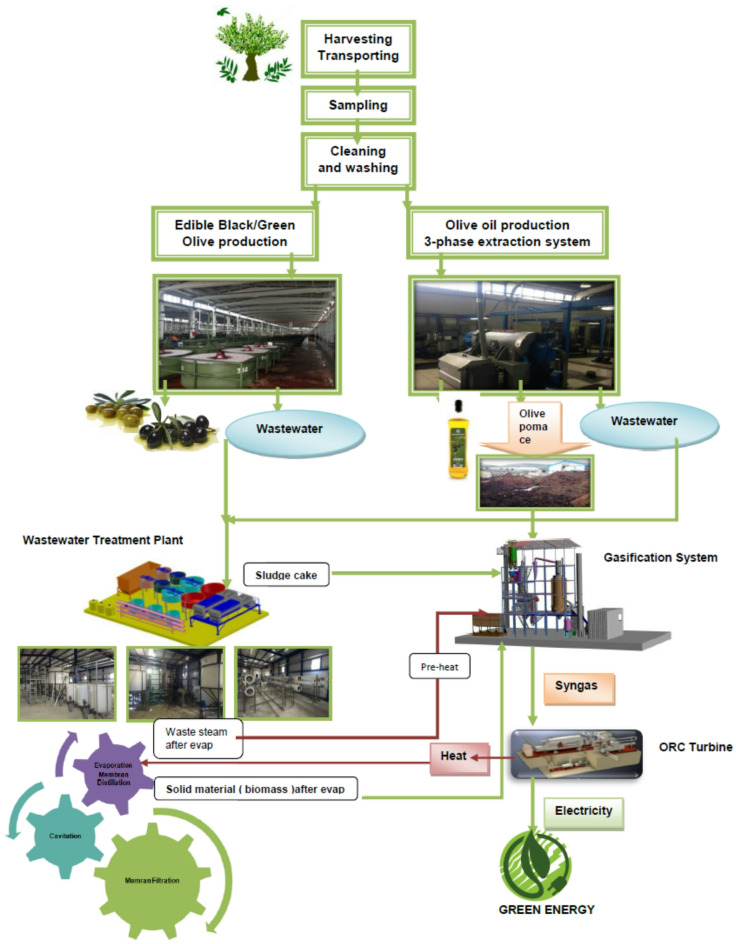
OMW management flow diagram.

In this study, olive production wastewaters were treated using an innovative integrated management approach that enabled the recovery of water, salts, and energy. Following preliminary physicochemical pretreatment, the wastewater underwent advanced treatment through cavitation and membrane filtration processes. The resulting concentrate was further processed via evaporation or membrane distillation. The final concentrate sludge was co-processed with olive pomace biomass as an energy source in the energy production stage (Fig. 1).

### Feedstock and system components

The primary feedstock used in this study was olive pomace, a solid residue from olive oil production. To ensure efficient gasification, the pomace was preconditioned to a suitable particle size and moisture content, such as in pelletized form. The integrated system consisted of a biomass gasification unit and an ORC turbine that utilizes the hot syngas produced to generate electricity. In the gasification unit, olive pomace undergoes partial combustion at high temperatures to form a combustible gas mixture while producing a solid by-product—biochar—rich in unburned carbon. Simultaneously, the syngas exiting the gasifier is combusted in a chamber to provide thermal energy to the ORC system.

**Fig. 2:**
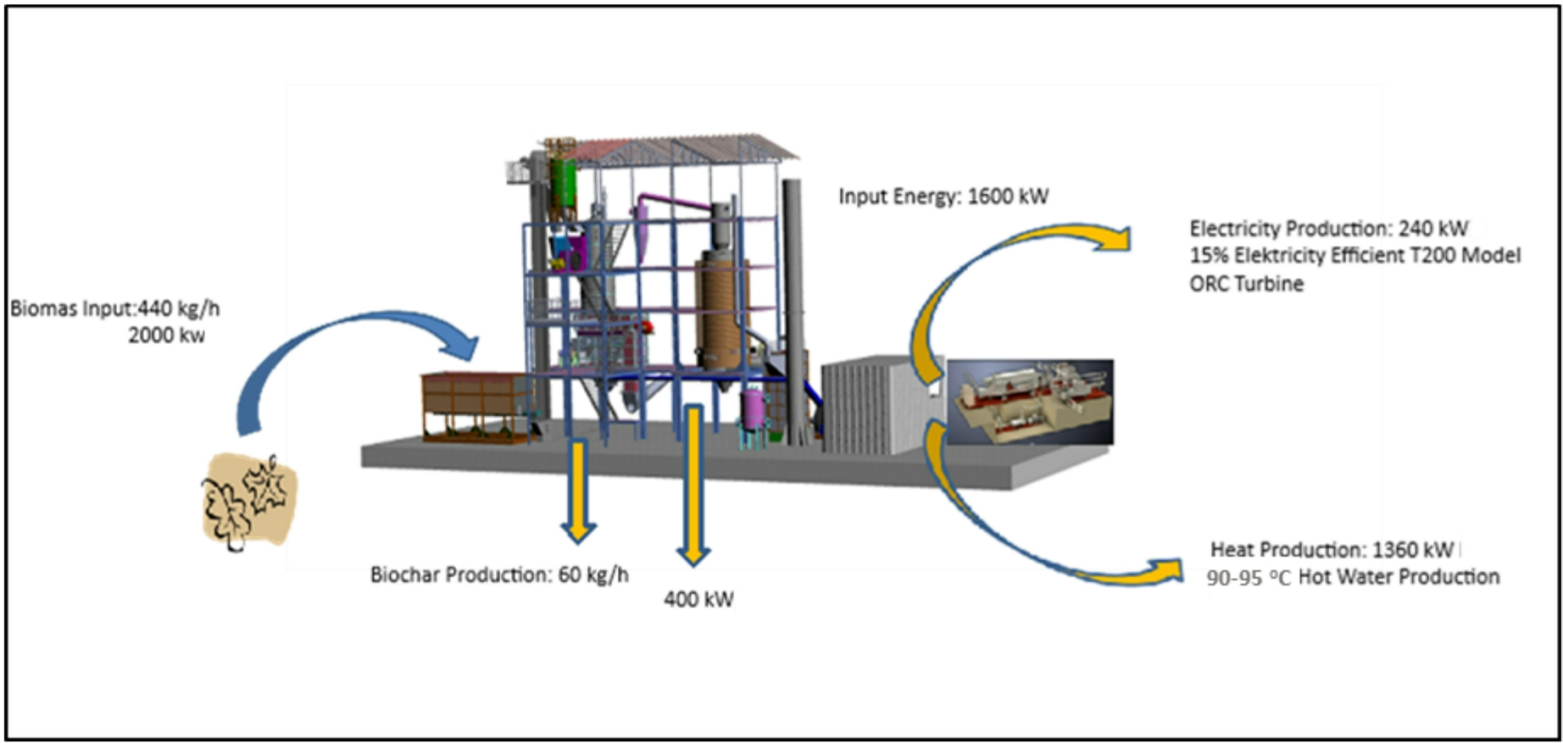
Sustainable bioenergy system summary.

The ORC turbine, designed to operate with an organic working fluid, vaporizes and expands the fluid to generate approximately 240 kW of electricity. The ORC system was selected due to its high efficiency in converting medium-temperature heat sources (e.g., gasifier flue gas or hot water/oil loops) into electricity, particularly in small- and medium-scale facilities (Fig. 2).

### Heat recovery and zero liquid discharge (ZLD) process

The integration of gasification and ORC results in considerable residual heat after electricity generation. To recover this heat, a heat exchanger system was designed to produce hot water at 90–95 °C. This hot water was used to evaporate olive mill wastewater from the olive oil facility. A pilot-scale evaporation system was successfully designed and installed to enable thermal treatment and water recovery from the wastewater. The system consists of a feed tank, an evaporation tank, a circulation pump, a tubular heat exchanger, and a set of control and measurement valves.

The superheated thermal oil exiting the ORC turbine at 315 °C served as the primary heat source. It was cooled to approximately 255 °C after transferring heat to both the olive mill wastewater and the membrane concentrate via a tubular heat exchanger. Inside the evaporation tank, the wastewater was evaporated at 108–114 °C, reducing its volume and concentrating the organic load. The majority of the water in the wastewater was recovered through condensation, while the remaining organic and inorganic components became concentrated. This recovery process ensured that the facility discharged no liquid waste, thereby achieving the goal of zero liquid discharge.

Importantly, the latent heat of the generated steam was not wasted. It was recovered and redirected to the biomass gasification reactor to enhance syngas production and improve the overall thermochemical efficiency. This approach contributed significantly to system integration and energy optimization.

### System performance and economic evaluation

A mass and energy balance analysis was conducted to evaluate the performance of the integrated system. All inputs—including pomace feed rate, moisture content, and lower heating value—and outputs—including syngas, biochar, flue gas, and hot water—were identified to ensure mass and energy conservation within the system boundaries. Based on these balances, key technical indicators were calculated, including gasification efficiency, biochar yield (both by mass and energy content), and ORC electricity generation efficiency.

The quantities of electricity (kWh), thermal energy, water (m³), and biochar (tonnes) produced per hour were determined. For the economic assessment, production costs and revenues were compared. Operating costs were itemized and calculated monthly, including feedstock procurement (e.g., processing, pelletizing, transportation), auxiliary energy (e.g., start-up fuel, internal electricity use), labor (e.g., plant operators), maintenance and repair, and depreciation/rent costs (especially for the ORC turbine). Two revenue scenarios were considered: (1)On-site consumption of electricity to reduce grid demand (interpreted as cost savings), (2) Full sale of generated electricity to the grid. Revenue from the sale of biochar, a valuable by-product, was also included. Furthermore, alternative biomass feedstocks such as corn cobs and wood chips—common agricultural residues—were analyzed in the cost model to account for seasonal fluctuations in pomace availability outside of the olive harvest season.

## Results

### Mass and energy balance

The gasification + ORC system, which uses biomass as feedstock, is based on biomass gasification and features an integrated tri-generation structure comprising electricity, heat (hot water), and biochar production. During the gasification process, the syngas generated from biomass is converted into electricity via the ORC system, while the unconverted carbon-rich solid residue is retained as biochar. This enables the simultaneous production of energy and biochar.

The calculated mass and energy balance of the integrated system demonstrates that a significant portion of the olive-based biomass can be converted into usable energy. For a biomass feed rate of 440 kg/h, the system generates approximately 240 kW of net electricity. This corresponds to about 12% of the pomace’s thermal energy content (approximately 2000 kW) being converted into electrical energy. The amount of biochar produced during the gasification process is estimated at 60 kg/h, meaning that about 14% of the input biomass is converted into a stable solid product by weight. This biochar is rich in carbon and minerals and accounts for approximately 20% of the feedstock’s energy content, corresponding to roughly 400 kW of thermal energy. The residual heat energy from the conversion process reaches approximately 1360 kW, most of which is available in the form of hot water at around 90 °C. Simultaneously, the combustible syngas generated in the gasifier is burned in a combustion chamber to supply thermal energy to the ORC system. Table 1 presents the mass and energy distribution of the ORC system, including biomass input characteristics, energy conversion stages, and associated system losses.

**Table 1:** Mass and energy balance of the biomass-fueled ORC system.

Stage	Value
Wet biomass feed (15% moisture)	439.82 kg/hour
Dry biomass feed (0% moisture)	373.85 kg/hour
Calorific value of dry biomass (kWh/kg)	5.35 kWh/kg
**Total energy input**	**2000 kW**
Gasifier + Boiler + Flue losses (20%)	400 kW
Ash and biochar losses (10%)	200 kW
Thermal input to ORC system	1600 kW
Electrical output from ORC (15% efficiency)	240 kW
ORC cooling losses (waste heat, 80%)	1280 kW
ORC thermal losses (5%)	80 kW
**Overall system electrical efficiency**	**12%**

The mass and energy balance presented here is based on steady-state operation of the pilot-scale system. Key assumptions include a biomass feed rate of 440 kg/h, an average moisture content of 15%, and a lower heating value (LHV) of 5.35 kWh/kg for dry biomass. The system boundaries cover all stages from biomass input and gasification to syngas combustion and electricity generation via the ORC unit, including thermal and flue losses. Overall electrical efficiency was calculated as 12%, which aligns with typical values for small-scale ORC systems operating on biomass-derived heat. In contrast, conventional anaerobic digestion combined heat and power (AD→CHP) systems generally reach 30–40% electrical efficiency and 70–90% overall efficiency when waste heat is recovered. This comparison highlights that, while the gasification–ORC configuration has lower direct electrical yield, it compensates through multi-product valorization—namely electricity, biochar, and recovered water—under a zero-discharge framework.

ORC systems are widely preferred in small- to medium-scale facilities, particularly for recovering heat from low- to medium-temperature sources such as gasifier flue gases or hot water/oil loops. This preference stems from their simplicity, relatively low cost, and efficiency advantages at smaller operational scales^27^. Calculations indicate that the available thermal energy in the system is sufficient not only to evaporate the inherent moisture content of the olive pomace but also to treat additional OMW through thermal evaporation.

This approach aligns with the principles of zero liquid discharge (ZLD) and circular economy strategies, offering a sustainable solution for the valorization of agro-industrial wastewater streams^28,29^. Furthermore, the recovery of latent heat via ORC systems has been shown to significantly improve the overall energy efficiency of integrated waste treatment facilities^30^. Previous studies on biomass valorization have also confirmed the practicality of combining gasification with steam recovery, demonstrating its effectiveness in enhancing syngas yield while minimizing environmental discharges^1,4^.

According to the analysis results (Table 2), the lowest total monthly cost, calculated as $26,133.04, was observed for corn cob. In this context, corn cob presents a cost-effective option for facilities that aim to minimize operational expenses. However, this economic advantage must be evaluated alongside its technical drawbacks, particularly its low calorific value and high ash content, which may impact system performance and maintenance requirements.In comparison, the total monthly cost for pelletized olive pomace was determined to be $31,147.32, of which $16,045.71 corresponds to raw material expenses. Despite having a relatively higher cost, olive pomace is valuable from a sustainability perspective, as it allows for the recovery of energy from olive industry waste. Additionally, its pelletized form offers benefits such as more uniform combustion, ease of storage, and compatibility with automated feeding systems. These characteristics make olive pomace a viable feedstock, despite its cost disadvantage.

Wood chips represent the most expensive option, with a total monthly cost of $32,439.91, of which $17,338.29 is attributed to raw material costs. Wood chips are compatible with many biomass conversion technologies and benefit from a broad supply network. However, their high procurement cost reduces their economic competitiveness. Nonetheless, their low moisture content, consistent supply, and compatibility with standard combustion systems may make them preferable under certain operational conditions.In summary, when considering total monthly operating costs, corn cob emerges as the most economical feedstock, followed by pelletized olive pomace and wood chips. However, feedstock selection should not be based solely on cost. Instead, it must be assessed through multi-dimensional criteria such as energy output per unit mass, emission profile, supply chain reliability, and technological compatibility.

**Table 2:** Monthly cost comparison of biomass feedstocks for 240 kW electricity generation.

Raw material	Raw material cost ($/m)	Electricity ($/m)	Gas ($/m)	Personnel ($/m)	Maintenance/other ($/m)	Total cost ($/m)
Corn Cob	11,031.43	4,132.69	4,397.49	6,428.57	142.86	26,132.04
Pelletized olive pomace	16,045.71	4,132.69	4,397.49	6,428.57	142.86	31,147.32
Wood chips	17,338.29	4,132.69	4,397.49	6,428.57	142.86	32,439.91

### Profitability comparison under electricity sales scenarios

This biomass-based energy production system was evaluated from both technical and economic perspectives, with a focus on revenue generation through electricity and biochar production. With an ORC turbine operating at 15% efficiency, the system generates 149,760 kWh of electricity per month from 440 kg/h of biomass feed (based on 26 days of continuous operation at 24 h/day). In an alternative scenario with 20.6% efficiency and 330 kW capacity, monthly production increases to 205,920 kWh.

Electricity generation capacities of 240 kW (base scenario) and 330 kW (efficiency-enhanced scenario) were evaluated under two marketing strategies: on-site consumption (internal use) and grid sales (external sales). Assuming a unit price of $0.12/kWh for internal use and $0.29/kWh for external sales, monthly electricity revenues were calculated. Internal sales yield revenues of $17,757.26 (240 kW) and $24,416.23 (330 kW), while external sales generate $43,697.83 (240 kW) and $60,084.51 (330 kW).

These findings clearly indicate that grid sales provide 2.5 to 3 times more revenue than on-site consumption for the same amount of energy produced. This underscores the economic impact of feed-in tariffs and supportive energy policies for renewable energy. However, practical considerations such as technical feasibility (grid connection capacity, reliability), legal restrictions, and the facility’s own energy demand must also be taken into account.

### Biochar revenue scenarios

In this integrated biomass utilization system, a multi-scenario revenue analysis was conducted for biochar commercialization in different forms—powder, briquette, and hookah charcoal—alongside electricity generation. All scenarios are based on a fixed biochar production rate of 0.1 ton/hour and continuous 24-hour operation for 26 days/month. Monthly revenues for the three biochar forms were calculated as follows: powder biochar ($142.86/ton) yields $8,914.29/month, briquetted biochar ($1,785.71/h) yields $111,428.57/month, and hookah charcoal ($3,214.29/h) yields $200,571.43/month.

These results show that while powdered biochar generates basic income, value-added products like briquettes and hookah charcoal can increase revenue by 10 to 20 times. This highlights the importance of product diversification, market segmentation, and downstream processing capacity in achieving economic sustainability in biochar enterprises.

### Integrated assessment and strategic insights

**Fig. 3:**
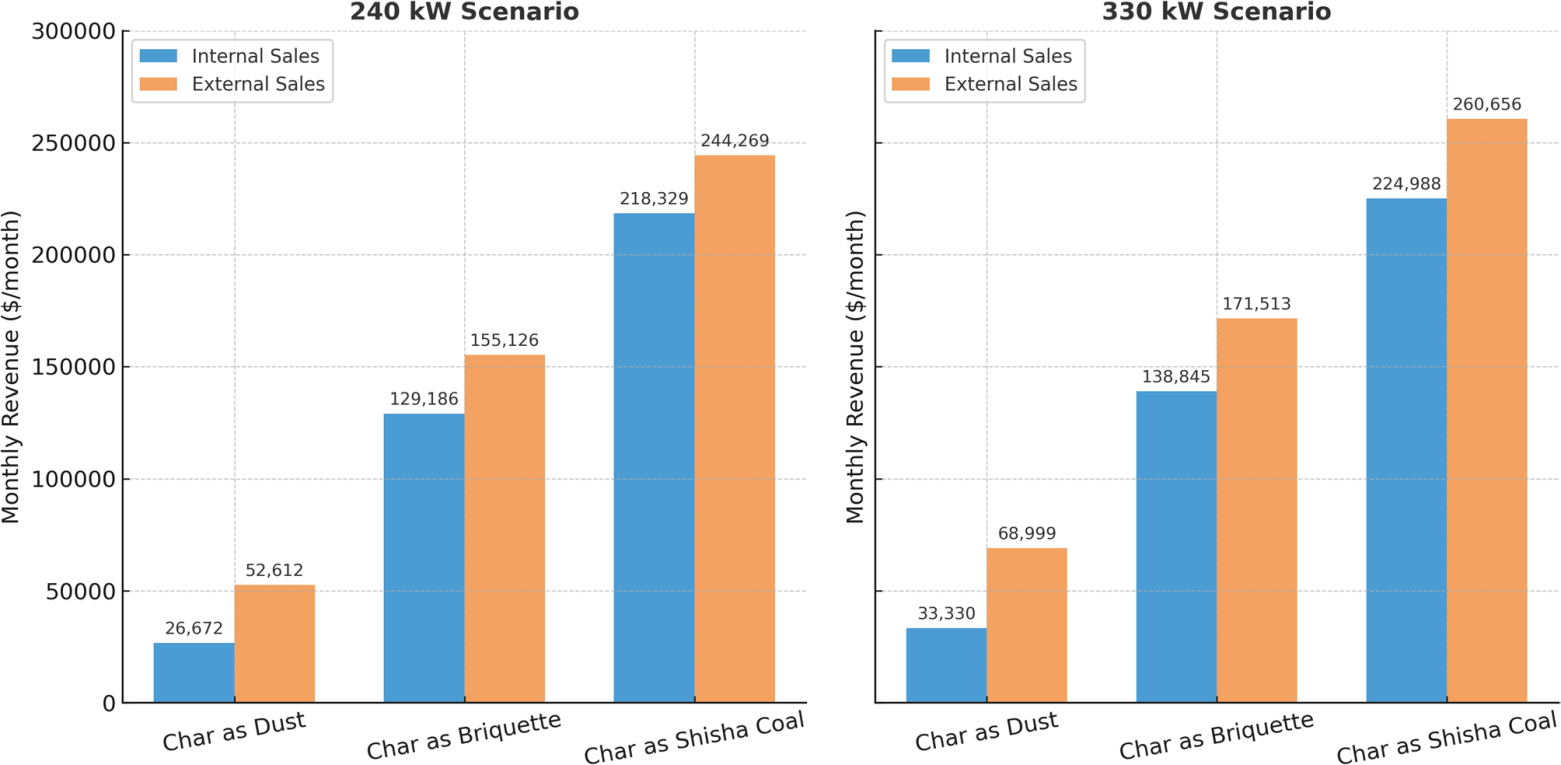
Monthly revenues for 240 kW and 330 kW scenarios showing the effect of biochar product form and electricity sales type on total income.

When both electricity and biochar revenues are considered, the results show that the form in which biochar is marketed significantly affects the overall economic performance of the energy system. As shown in Fig. 3, the type of biochar product and electricity generation capacity both influence monthly revenue.In the 240 kW scenario, electricity sales alone generate $17,757.26, while powdered biochar adds $8,914.29, for a total of $26,671.55—an indication that low-value products are insufficient to achieve high profitability. However, marketing biochar in briquette form increases monthly revenue to $129,185.83, and hookah charcoal raises it further to $218,328.69. These results show that the profitability of the project is highly dependent on the biochar product form. Hookah charcoal significantly boosts revenues due to its high market value.Under the external sales scenario, electricity revenue increases to $43,697.83, while biochar revenues remain unchanged. In this case, total monthly revenues are: $52,612.06 (powder), $155,126.26 (briquette), and $244,269.26 (hookah). The increase in electricity price (external sales vs. internal use) contributes more significantly to total revenue when biochar is of lower value. However, for high-value products like briquettes and hookah charcoal, biochar remains the dominant revenue stream.

A similar trend is observed in the 330 kW scenario. With electricity revenue rising to $24,416.23, a 38% increase is achieved. Combined monthly revenues reach $33,330.50 with powder biochar, $138,844.80 with briquetted biochar, and $224,987.66 with hookah charcoal. Hence, upgrading biochar into high-value forms contributes far more to total income than improving electricity output.In the highest income scenario (330 kW + external sales), electricity revenue reaches $60,084.51. Total monthly revenues are $68,998.88 for powdered biochar, $171,513.03 for briquetted biochar, and $260,655.94 for hookah charcoal. These results demonstrate that as energy capacity and marketing strategy improve, the financial contribution of value-added biochar becomes increasingly significant. In all scenarios, biochar revenues accounted for 70–90% of the total income, indicating that biochar is not a by-product, but a central element of the system’s financial sustainability.

Revenue sources such as waste heat recovery, carbon credit sales, or thermal energy sales to drying systems were not included in this analysis due to a conservative approach. Thus, the profitability estimates presented here reflect the most cautious scenarios. With appropriate business models and policy support, the economic performance of the system can be further improved. Future studies are recommended to evaluate these systems holistically, incorporating environmental and economic indicators such as cost-benefit ratios, carbon offset credits, and life cycle emissions. Scenario-based modeling allows flexible adaptation to market conditions, policy shifts, or resource availability.

## Discussion

The findings of this study demonstrate that olive processing waste can be fully utilized through an integrated approach, proving the practical applicability of the “waste-to-resource” principle. The proposed system enables the management of both solid and liquid waste streams generated during olive oil production without any discharge into the environment. This system was developed and tested at pilot scale, under real operating conditions in an olive oil production facility, confirming its practical applicability beyond laboratory conditions. In the integrated process, olive pomace is converted into fuel and thus eliminated, while the water content in olive mill wastewater is evaporated and condensed for recovery. This outcome represents a significant gain, particularly in addressing water scarcity, which is frequently experienced in the Mediterranean basin. The recovered pure water can be reused as an additional source for agricultural irrigation^31^. Although the study was conducted at pilot scale, further scale-up applications would strengthen the finding. Large-scale operation may introduce additional challenges related to system integration, operational stability, and continuous feedstock supply. Therefore, validation and scale-up studies are essential before the system can be considered a fully proven industrial option. The pilot plant occupies a total area of approximately 400 m², consisting of a gasifier unit (10 m width × 30 m length × 12 m height) and an ORC turbine hall (6 m width × 12 m length × 6 m height). These dimensions reflect an intermediate-scale installation that bridges laboratory demonstration and full industrial implementation.

Beyond the AD→CHP baseline, alternative pathways may be preferable depending on site conditions. Gasification coupled to an internal-combustion engine (gasification→ICE) can deliver higher electrical output at small/medium scale when syngas cleanup and tar control are robust (tar typically < ~ 50 mg/Nm³)^32,33^. In contrast, direct biomass combustion with a steam Rankine cycle becomes attractive mainly at larger thermal capacities and with reliable district-heating sinks^34–36^. Biogas upgrading to biomethane (RNG) reduces exposure to electricity tariffs where grid access and incentives exist^37,38^. For the AD→CHP baseline itself, biogas-fired reciprocating engines typically achieve ~ 30–42% electrical efficiency on an HHV basis, with overall (electricity + useful heat) efficiencies ~ 65–85% when heat is recovered^39–42^. For ORC pathways, typical net electrical efficiency is ~ 15–20%, with ~ 17% demonstrated around the 1 MW scale^43,44^. Technology choice should align with boundary conditions: wet substrates with dependable heat demand favor AD→CHP; access to premium biochar markets and stringent water-management constraints favor gasification–ORC; reliable gas grids and incentives favor biomethane; and large-scale sites with district-heating opportunities favor direct combustion with steam cycles.

The work of Dutournié et al.^5^ supports this approach, showing that olive mill wastewater can be dried and concentrated using appropriate methods to recover up to 95% of its water, which can then be rendered suitable for irrigation with basic treatment. Similarly, the integrated system developed in this study almost entirely recovers the wastewater, preventing environmental pollution and contributing positively to the regional water cycle. In traditional practices, the evaporation of this wastewater can take years and poses environmental risks during the process. In contrast, the proposed system eliminates the wastewater load rapidly. As a result, the “zero discharge” target is fully achieved, completely preventing the discharge of wastewater from olive processing facilities into receiving environments. For completeness, AD of olive mill residues is technically feasible, yet olive mill wastewater (OMWW) often hampers digestion stability due to phenolic toxicity, and performance gains are frequently reported when co-digestion or adsorptive supports (e.g., biochar) are employed^45–47^.

The energy outputs and by-products obtained demonstrate that olive waste can be effectively valorized within the framework of green energy and circular economy. The electricity generated can meet the internal energy needs of the olive oil facility, reducing dependence on external energy sources, or it can be sold to the grid, contributing to renewable energy supply. Thus, with appropriate technologies, olive waste can be transformed into a renewable energy source. Biochar, a solid by-product, has significant value-added potential. As a high-carbon material, biochar can be used in agriculture as a soil conditioner. Literature reports that biochar derived from olive waste promotes root development and increases the water and nutrient retention capacity of soils. Additionally, its alkaline nature and mineral content may help regulate soil pH and adsorb pollutants such as heavy metals, thereby contributing to environmental health. The carbon content in biochar is in a recalcitrant form, meaning it remains stable in the soil for long periods. This implies carbon sequestration, effectively removing CO₂ from the atmosphere and storing it in the soil^4^.

Thus, the valorization of olive waste as biochar not only ensures waste disposal but also creates a cycle that contributes to agricultural production and carbon sinks. Such applications can enhance economic returns. For example, high-quality olive pit charcoal can meet local demand for barbecue charcoal, offering a domestic alternative to imported products. Similarly, biochar can be processed into activated carbon for use in water and wastewater treatment. In this context, when olive processing facilities begin to manage their waste through integrated systems, a new income stream and business line can emerge—particularly contributing to economic development in rural areas. Consequently, the present pathway is biochar-led in value creation, whereas AD→CHP systems are electricity/heat-led, underscoring a product-portfolio rather than single-metric (η_el) comparison^42,48–50^.

The economic feasibility of the integrated system was further evaluated using pilot-scale operational data to quantify investment performance. The gasifier–ORC configuration (0.5 t h⁻¹; T200-CHP) entails a total capital expenditure (CAPEX) of approximately 678,000 USD—comprising 428,000 USD for the gasifier unit and 250,000 USD for the ORC turbine. Assuming continuous operation of 8,000 h year⁻¹, the system yields a net annual profit of about 180,000 USD when fueled with corn-cob biomass, corresponding to a simple payback time of 3.8 years. For other feedstocks, annual profits and payback times were estimated as 130,000 USD (5.1 years) for woodchips and 116,000 USD (5.9 years) for olive-pomace pellets.These estimates represent net profits after accounting for feedstock, labor, maintenance, ignition gas, and internal electricity costs, providing a realistic first-order profitability picture. Importantly, the above figures exclude revenues from recovered thermal energy (hot water at 90 °C and vapor at 114 °C). When this waste heat is valorized—such as via evaporation or district heating—the overall profitability improves by approximately 50%, reducing the payback period to around 2.5 years.

Under current electricity and fuel prices, renewable-energy incentives remain essential for reaching the break-even point more rapidly. Moreover, the system eliminates waste-disposal costs that olive producers would otherwise bear, adding a “hidden gain” to the financial balance of operations. Nonetheless, establishing a gasification–ORC facility requires significant upfront investment and technical expertise. Hence, public–private cooperation and appropriate financial mechanisms (e.g., credits, feed-in tariffs) would accelerate adoption and strengthen economic viability^51,52^.

Another important discussion point is the seasonality of olive waste supply. Olive harvesting is concentrated in certain months of the year, meaning feedstock availability may be insufficient for full-capacity operation year-round. This issue can be addressed by drying and pelletizing the pomace during the harvest season and storing it for use in the remaining months. Alternatively, other regional biomass wastes (such as corn cobs, pruning residues, and wood chips) can be included in the fuel portfolio of the facility. Economic analyses conducted in this study have shown that similar energy production potential can be achieved with alternative feedstocks such as corn cobs and wood chips. This allows the facility to remain operational beyond the olive season and increases the efficiency of the investment. However, the use of different feedstocks can complicate supply chains and preprocessing operations, making optimal planning based on local conditions essential. From the conversion side, ORC electrical efficiencies vary widely with working fluid, expander type, and heat-source quality, which motivates site-specific optimization when supplementing olive residues with regional biomass^50–54^.

From an environmental perspective, the proposed integrated system offers the most ecological solution for managing olive waste. The fact that wastewater is not discharged into receiving environments means that soil and water pollution loads are reduced to zero. Solid waste, being combusted under controlled conditions and converted into biochar, results in significantly lower greenhouse gas emissions compared to open burning or decomposition scenarios. Energy derived from biomass leads to lower net GHG emissions compared to fossil fuel-based energy. The CO₂ emitted from the combustion of olive tree residues is considered carbon-neutral, as it was originally sequestered from the atmosphere during the tree’s growth^1^. In this respect, the integrated system can even be regarded as a negative emission technology (biological carbon capture and storage). Ultimately, the use of olive processing waste in this way not only mitigates local environmental problems (e.g., water/soil pollution, odor, waste accumulation) but also represents a climate-friendly approach on a global scale. n parallel, AD digestate can be reused as a soil amendment/fertilizer, often with favorable agronomic and environmental profiles when appropriately managed, which further motivates side-by-side environmental and agronomic assessments of the two pathways^54,55^.

## Conclusion

This study demonstrates that olive processing waste can be effectively managed through an integrated gasification–ORC system aligned with the zero-waste principle, enabling the recovery of clean water and the generation of green energy. Nearly all olive waste can be valorized: wastewater is treated to recover pure water, pomace is converted into electricity and heat, and the residual biochar can be used as fertilizer or fuel. Thus, olive oil facilities can become self-sufficient energy producers and reduce their reliance on external waste disposal. A facility processing approximately 440 kg/h of pomace can generate 240 kW of continuous renewable electricity, helping offset CO₂ emissions and recover thousands of cubic meters of water annually. The pilot-scale implementation confirmed that the zero-discharge target is both technically and economically feasible. Key recommendations include the need for supportive policies and financial mechanisms such as renewable energy incentives, carbon credits, and investment grants to promote adoption. Cooperative waste facilities can provide scale advantages and reduce individual costs, while public–private partnerships should support pilot projects and technology dissemination. Standardizing and commercializing biochar, along with its agricultural use as a soil amendment, should be encouraged.

In conclusion, olive waste can be transformed from an environmental burden into an economic asset. The zero-discharge, green energy model offers a viable route for promoting sustainability and circular economy in the olive oil sector. With broader application to other agricultural wastes, this approach could support integrated waste management and renewable energy production across the agri-food industry. Although the proposed system demonstrates promising technical and environmental results, its economic feasibility strongly depends on supportive policy frameworks, stable biochar markets, and technology scale-up. Further pilot-scale validation and long-term field demonstrations are required to confirm the feasibility of this pathway under real operating conditions.

## Data Availability

The datasets used and/or analysed during the current study available from the corresponding author on reasonable request.
